# Optimizing the Limit of Detection of Waveguide-Based Interferometric Biosensor Devices

**DOI:** 10.3390/s19173671

**Published:** 2019-08-23

**Authors:** Jonas Leuermann, Adrián Fernández-Gavela, Antonia Torres-Cubillo, Sergio Postigo, Alejandro Sánchez-Postigo, Laura M. Lechuga, Robert Halir, Íñigo Molina-Fernández

**Affiliations:** 1 Bionand Center for Nanomedicine and Biotechnology, Parque Tecnológico de Andalucía, 29590 Málaga, Spain; 2 Department de Ingeniería de Comunicaciones, Universidad de Málaga, ETSI Telecomunicación, Campus de Teatinos, 29071 Málaga, Spain; 3 Departamento de Física, Universidad de Oviedo, C/Federico García Lorca, 33007 Oviedo, Spain; 4 Department de Ingeniería Mecánica, Universidad de Málaga, Térmica y de Fluidos, Escuela de Ingenierías Industriales, Campus de Teatinos, 29071 Málaga, Spain; 5 Nanobiosensors and Bioanalytical Applications Group, Catalan Institute of Nanoscience and Nanotechnology (ICN2), CSIC, BIST and CIBER-BBN Campus UAB, 08193 Barcelona, Spain

**Keywords:** limit of detection, coherent detection, silicon photonics, interferometer, biosensors

## Abstract

Waveguide-based photonic sensors provide a unique combination of high sensitivity, compact size and label-free, multiplexed operation. Interferometric configurations furthermore enable a simple, fixed-wavelength read-out making them particularly suitable for low-cost diagnostic and monitoring devices. Their limit of detection, i.e., the lowest analyte concentration that can be reliably observed, mainly depends on the sensors response to small refractive index changes, and the noise in the read-out system. While enhancements in the sensors response have been extensively studied, noise optimization has received much less attention. Here we show that order-of-magnitude enhancements in the limit of detection can be achieved through systematic noise reduction, and demonstrate a limit of detection of ∼$10^{-8}\;\mathrm{RIU}$ with a silicon nitride sensor operating at telecom wavelengths.

## 1. Introduction

Photonic integrated biosensors have been the subject of intense research in the last decade due to their capability to detect small quantities of biochemical substances such as protein biomarkers, DNA or toxins, indicative of the presence of a disease or environmental pollution without time-consuming labeling steps [1,2,3]. Indeed, lab-on-chip and point of care devices based on such sensors have been proposed for a variety of applications, including environmental safety, food control, and clinical diagnosis [4,5,6]. Realizing such biosensors in silicon platforms enables dense, multiplexed operation while CMOS fabrication compatibility minimizes costs [7]. The basic physical variation detected by photonic biosensors is a change in refractive index. Consequently, the sensitivity of such sensors is often expressed as the rate of change of the output signal per refractive index unit (RIU). Analogously, the limit of detection (LOD) is given as the smallest refractive index change that can be reliably detected. Photonic sensors only become specific to a certain analyte by a proper biofunctionalization protocol of the corresponding selective bioreceptors on the sensor surface. In order to detect very low concentrations of a certain analyte, even when the analyte is present in a complex medium, both a highly selective biofunctionalization able to generate an antifouling sensor surface and a very good photonic LOD are thus required. Since the biofunctionalization is inherently application dependent, optimizing the photonic sensor LOD is usually the first step in a sensor development. The LOD is given by LOD=3σ/S in refractive index units, where σ is the system noise, and *S* is the sensitivity. An improvement in both sensitivity and noise will thus result in enhanced limits of detection.

In photonic waveguide sensors light interacts with the analyte via the evanescent tail of the waveguide mode. The waveguide sensitivity, $S_{\mathrm{wg}}$, is defined as the change in the mode effective index resulting from a change in the refractive index of the medium surrounding the waveguide. The mode effective index change is then transduced into a measurable quantity such as a change in output power or resonance wavelength via the sensing architecture, e.g., a Mach-Zehnder interferometer (MZI) or a ring resonator. The overall sensitivity depends both on the waveguide sensitivity and the specific sensing architecture [1,8,9]. The waveguide sensitivity can be significantly enhanced by using slot-waveguides [10,11], subwavelength gratings (SWG) [2,12,13,14,15,16,17,18,19], or by using transverse-magnetic (TM) instead of transverse-electric (TE) modes [20], with values up to $S_{\mathrm{wg}}∼\;0.8\;\mathrm{RIU}/\mathrm{RIU}$. Two different sensing architectures are widely used in photonic biosensing: resonant and interferometric arrangements [21]. Single rings or multiple rings using the Vernier effect have been shown to reach state-of-the-art LODs of around $10^{-6}\;\mathrm{RIU}$ [14,16,17,22,23,24]. Unfortunately, to read the sensor signal they require a tunable laser source or a white light source and a spectrum analyzer [23], thereby increasing the complexity of the overall sensing system. Interferometric Mach-Zehnder sensors, conversely, only require a fixed wavelength source and direct power detection at the output. While basic Mach-Zehnder configurations suffer from sensitivity fading and phase ambiguity [25], different techniques have been proposed to overcome this limitation, e.g., wavelength [26] and thermal [27] modulation. Coherently detected interferometers provide completely linear phase read-out [28,29], offering state-of-the-art bulk LOD [22] and calibration techniques than can cancel hardware imperfections [30]. Most recent MZI-based sensors report LODs of the order of $10^{-7}\;\mathrm{RIU}$ [4,11,18,21,22,26,27,31,32]. An exceptionally low LOD of $2.7\times 10^{-8}\;\mathrm{RIU}$ was reported in [22], albeit using an imbalanced silicon MZI with a 9mm length difference, which exhibits a stronger sensitivity to laser phase jitter than a balanced interferometer.

**Figure 1:**
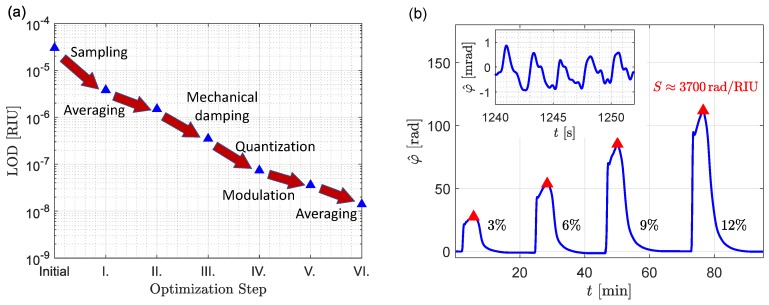
(**a**) Measured limit of detection of the integrated waveguide-based sensing system as different noise sources are addressed. (**b**) Sensor calibration with NaCl solutions shows a sensitivity of ≈3700 rad/RIU. The reduced noise shown in the inset is only achieved after dampening of mechanical noise and 2Hz low-pass filtering (0.5s averaging), i.e., steps I–III in Figure 1a.

Read-out noise reduction has received much less attention than sensor optimization, despite its significant impact on the LOD [8,33,34]. Previous theoretical efforts have shown that interferometric sensors can reject amplitude and phase noise of the laser source, so that detection becomes fundamentally limited by thermal and shot noise, as well as intrinsic waveguiding losses [8]. Here we provide a holistic approach for LOD optimization through which this fundamental limit can be approached in practical sensing systems. Specifically, we show how by systematic experimental characterization of mechanical and electrical noise sources (shot noise, amplifier noise and quantization noise) and subsequent read-out optimization, the LOD can be enhanced significantly, as illustrated in Figure 1a. With the proposed procedure we are able to demonstrate a bulk LOD of $1.4\times 10^{-8}\;\mathrm{RIU}$ with a 5s averaging time, using a balanced MZI with 6mm long silicon nitride waveguides that exhibit a comparatively low sensitivity $S_{\mathrm{wg}}∼0.2\;\mathrm{RIU}/\mathrm{RIU}$.

The paper is organized as follows. In Section 2 (Methods) we theoretically analyse the sensitivity and the noise sources in an interferometric biosensing setup and quantify their impact on the limit of detection. In this section we also describe the equipment and the procedure to measure the LOD. A practical guide to LOD enhancement, addressing both mechanical and electrical noise, is presented in Section 3, together with the experimental validation of the LOD improvement. Finally, in Section 4, conclusions are drawn.

## 2. Methods

In this section we present the sensitivity of the interferometric sensors (Section 2.1), analyse the different noise sources (Section 2.2) and study their impact on the LOD (Section 2.3). In Section 2.4 the experimental procedures are described.

### 2.1. Sensitivity

**Figure 2:**
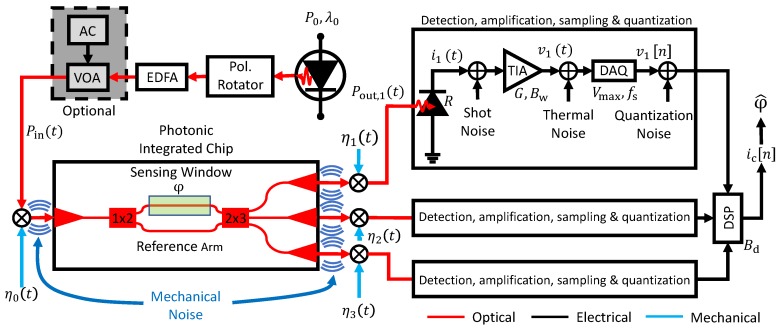
Setup of the employed interferometric sensing system, with mechanical and electrical noise sources. Mechanical vibrations result in time dependent coupling efficiencies η(t) to/from the sensor chip, while photo-detection, amplification, sampling and quantification add shot noise (SN), thermal noise (TN), aliasing and quantization noise (QN), respectively. The presence of noise hinders the accurate estimation ($\hat{\varphi }$) of the sensed phase shift (φ) resulting in a deterioration of the limit of detection.

The interferometric sensing setup shown in Figure 2 is considered in the following. Note that although a coherent 2×3 read-out is used for linear phase read-out [28,29,31,32], the techniques we outline are equally applicable for a single photodiode read-out. A laser emits light with wavelength $\lambda_{0}$ and power $P_{0}$, rotated to horizontal polarization. After amplification in an erbium doped fiber amplifier (EDFA) and optional modulation via a variable optical attenuator (VOA) light with mean power ${\bar{P}}_{\mathrm{in}}$ couples into the photonic sensor chip. Light injection into the chip is performed by fiber-to-chip surface grating couplers with a coupling efficiency $\eta_{0}$. The sensor chip itself consists of a balanced MZI with a sensing arm, exposed to the aqueous buffer containing the analyte, a reference arm, and a 2×3 multimode interference coupler (MMI); further details about the sensing chip are given in Section 2.4. A refractive index change in the buffer causes a phase shift $\varphi \left(t\right)=2\pi /\lambda_{0}L_{s}\Delta n_{\mathrm{eff},s}\left(t\right)$, where $L_{s}$ is the length of the reference and sensing waveguides and $\Delta n_{\mathrm{eff},s}$ is the change in the mode index due the presence of the analyte. The three different outputs of the MMI, k={1,2,3}, couple out of the chip with different coupling efficiencies $\eta_{k}$. The power $P_{\mathrm{out},k}$ at each output is then photo-detected, linearly converting the received optical power into a electrical current $i_{k}\left(t\right)$ with responsivity *R*. A consecutive transimpedance amplifier (TIA) linearly converts the current into a voltage $v_{k}\left(t\right)=Gi_{k}\left(t\right)$, with a certain gain *G*. A data acquisition board (DAQ) samples and quantizes the continuous signal into its digital representation, $v\left[n\right]=v\left(t=n/f_{s}\right)$, where *n* is an integer and $f_{s}$ is the sampling frequency. From the three photocurrents illustrated in Figure 2, digital signal processing computes the complex current $i_{c}\left(t\right)=I_{\mathrm{out}}\exp\left(j\hat{\varphi }\right)$ [28,29], where $I_{\mathrm{out}}\propto R{\bar{P}}_{\mathrm{out}}$ is its amplitude, *j* the imaginary unit and $\hat{\varphi }$ is an estimate of the optical phase shift φ. The overall sensitivity of the system is given by [8]

(1) $$S=\left(2\pi /\lambda_{0}\right)L_{s}S_{\mathrm{wg}}I_{\mathrm{out}}.$$

### 2.2. Noise Sources

Several noise sources appear in the detection process. Due to vibrations, the in- and out-coupling efficiencies will be time dependent, which results in a spurious amplitude modulation of the sensor signal, i.e., mechanical noise (MN). The detected photocurrent carries electrical shot noise (SN), and the amplification adds thermal noise (TN). The digitization of the sensor signal produces quantization noise (QN) and, possibly spectral aliasing. The noise in the system will generally depend on the power spectral density (PSD) of the noise sources and measurement bandwidth ($B_{d}$), or, equivalently, the integration time, i.e., $\sigma^{2}=\int_{B_{d}}\mathrm{PSD}\;df$. Referring all the noise sources to the input of the transimpedance amplifier, the power spectral densities of the different components of the noise current are given by

(2) $$\begin{array}{cc} {\mathrm{PSD}}_{\mathrm{SN}} & =2qR{\bar{P}}_{\mathrm{out}}\vartheta_{\mathrm{us}}, \end{array}$$

(3) $$\begin{array}{cc} {\mathrm{PSD}}_{\mathrm{TN}} & =R^{2}{\left[\mathrm{NEP}\left(G\right)\right]}^{2}\vartheta_{\mathrm{us}}, \end{array}$$

(4) $$\begin{array}{cc} {\mathrm{PSD}}_{\mathrm{QN}} & =\frac{\gamma^{2}}{G^{2}}\frac{2}{f_{s}}{\left(\frac{2V_{\max}}{2^{M}}\right)}^{2}, \end{array}$$

(5) $$\begin{array}{cc} {\mathrm{PSD}}_{\mathrm{MN}} & \propto {\bar{P}}_{\mathrm{in}}^{2}, \end{array}$$

where $q=1.6\times 10^{-19}\;C$ is the elementary charge, ${\bar{\eta }}^{2}$ is the mean of the product of the in- and out coupling efficiencies, ${\bar{P}}_{\mathrm{out}}\propto {\bar{\eta }}^{2}{\bar{P}}_{\mathrm{in}}$ is the mean received optical power of one photodiode, NEP is the noise equivalent power of the amplifier (which is normally dependent on the gain setting *G*), γ is the quality factor of the DAQ, $V_{\max}>v_{k}\propto G{\bar{\eta }}^{2}P_{\mathrm{in}}$ is the maximum input voltage of the DAQ, *M* is the number of quantization bits, $f_{s}$ is the sampling frequency, and $\vartheta_{\mathrm{us}}$ is a broadband noise under-sampling correction factor, $\vartheta_{\mathrm{us}}=2B_{w}/f_{s}$ with $B_{w}$ the bandwidth of the photodiode-amplifier system ($B_{w}>B_{d}$) [35]. This under-sampling factor is not relevant if a high-end DAQ card with integrated anti-aliasing filters is used. The expressions for shot and thermal noise were given in [8], while the expression for quantization noise is derived in Appendix A.1. The mechanical noise results in an amplitude modulation of the input signal, so that its power scales directly with the input power.

Note that the two-sided power spectral density of the noise signal can be computed directly from the measured signal v[n] via a Fourier-Transform,

(6) $$\mathrm{PSD}\left[f_{n}=nf_{s}/N\right]={\left|F\{v[n]\}\right|}^{2}/\left(Nf_{s}\right)\;\left[V^{2}/\mathrm{Hz}\right],$$

where F denotes the discrete Fourier transform [36], *N* is the number of samples, and *n* is an integer. This PSD this can be referred to the amplifier input by dividing by $G^{2}$.

### 2.3. Impact of Noise on the Limit of Detection

The overall noise is proportional to the squared sum of the individual noise contributions, and the sensitivity (see Equation (1)) is directly proportional to the output current, which in turn is proportional to the input power, so that the LOD is directly proportional to

(7) $$\mathrm{LOD}=\frac{3\sigma }{S}\propto \frac{\sigma }{{\bar{P}}_{\mathrm{in}}}\propto \sqrt{\left(\sigma_{\mathrm{SN}}^{2}+\sigma_{\mathrm{TN}}^{2}+\sigma_{\mathrm{QN}}^{2}+\sigma_{\mathrm{MN}}^{2}\right)/{\bar{P}}_{\mathrm{in}}^{2}}.$$

Writing out the individual factors and including only the terms that can be readily modified in the read-out, we find

(8) $$\begin{array}{cc} {\mathrm{LOD}}_{\mathrm{SN}}^{2} & \propto \frac{B_{d}}{{\bar{P}}_{\mathrm{in}}}\vartheta_{\mathrm{us}}, \end{array}$$

(9) $$\begin{array}{cc} {\mathrm{LOD}}_{\mathrm{TN}}^{2} & \propto \frac{{\left[NEP\left(G\right)\right]}^{2}}{{\bar{P}}_{\mathrm{in}}^{2}}\vartheta_{\mathrm{us}}B_{d}, \end{array}$$

(10) $$\begin{array}{cc} {\mathrm{LOD}}_{\mathrm{QN}}^{2} & \propto {\left(\frac{V_{\max}}{G{\bar{P}}_{\mathrm{in}}}\right)}^{2}\frac{B_{d}}{f_{s}}, \end{array}$$

(11) $$\begin{array}{cc} {\mathrm{LOD}}_{\mathrm{MN}}^{2} & \propto \int_{B_{d}}{\mathrm{psd}}_{\mathrm{MN}}\left(f\right)\;df, \end{array}$$

where ${\mathrm{psd}}_{\mathrm{MN}}$ is a function of frequency that only depends on the mechanical properties of the setup. From Equations (8)–(11) it is then clear that with increasing input power (${\bar{P}}_{\mathrm{in}}$) the impact of shot and thermal noise will be limited, while the impact of mechanical noise does not depend on the optical or electrical parameters of the setup. For a certain voltage range of the DAQ, the impact of quantization noise decreases with increasing signal power, and the best noise performance is achieved when the full range of the DAQ is used. However, if the signal is increased beyond the DAQ range (by either increasing the input power or the amplification), a wider range has to be chosen, resulting in a quadratic increase in quantization noise. A careful strategy is thus required to simultaneously minimize the impact of all noise sources.

### 2.4. Experimental Methods

**Figure 3:**
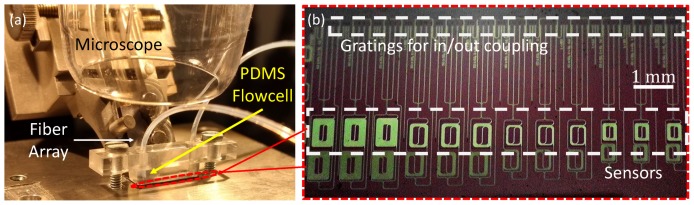
(**a**) Part of the experimental setup showing the fiber array for light coupling and the flowcell covering the photonic sensing chip. (**b**) Sensing chip highlighting the coupling zone for the fiber array and the sensor region.

The equipment used in the read-out system shown in Figure 2 consists of a Santec WSL-100 laser source, an IPG Photonics erbium doped fiber amplifier (EAD-500C) and the V1550A variable optical attenuator from Thorlabs for the generation of the input light. A custom fiber array from O/E Land is used for light coupling to and from the chip (see Figure 3a). At the output photo-detection and amplification is performed with the PDA10CS-EC from Thorlabs, and the signal is digitized using the USB-6210 DAQ by National Instruments. The main parameters of the devices are listed in Table A1 in the Appendix A.2. Processing of the three output signals is performed with Matlab. The sensing chip (≈5mm×10mm), based in a silicon wafer with 2.5μm BOX and 300nm silicon nitride film, was fabricated at the Instituto de Microelectrónica de Barcelona, Centro Nacional de Microelectrónica (IMB-CNM), CSIC [37] through a Multi Project Wafer approach offered by VLC Photonics. Twelve sensors in a row are integrated into the chip as shown in Figure 3b, each covering an area of approximately $0.4\;{\mathrm{mm}}^{2}$. The sensing waveguide was optimized for single-mode operation and low propagation losses, having a width of 1μm. Improving the waveguide sensitivity was not an objective and was not considered during the design process. The waveguide sensitivity was computed using the Fimmwave mode solver by Photon Design [38]: first, the effective index of the fundamental TE mode was calculated for a water cladding ($n_{H2O}=1.3162$ for a wavelength of 1.55μm); then, this effective index was recalculated for a small variation in $n_{H2O}$. A linear fit was used to extract the waveguide sensitivity. A PDMS flowcell containing a microfluidic channel with a width of 3mm, a length of 9mm, and a height of 0.5mm, is fixed on top of the sensing surface enabling a constant stream of the buffer media throughout the entire channel (see Figure 3a).

In order to identify different noise sources we examine the noise at one of the three output photo-detectors (see Figure 2). The potential impact of the noise source on the LOD is then estimated using Equations (8)–(11). The LOD is experimentally determined using a commonly used calibration procedure [22]. Four different sodium chloride (NaCl) solutions (0.5M, 1.0M, 1.5M, 2.0M) diluted in de-ionized, purified water (Milli-Q) are injected with a flow rate of 20μL/min. Since the sensor response is linear, and the sensitivity is thus independent of the specific refractive index change (see Equation (1)), the sensitivity does not depend on the concentrations for the evaluation. With the refractive indexes (*n*) of the solutions and their differences (Δn) to the buffer, shown in Table 1, calculated according to [39], a phase-to-refractive-index response curve can be determined as shown in Figure 1b. The shown data is available as part of the Supplementary Material.

Its slope represents the overall sensitivity in rad/RIU, i.e., $S/I_{\mathrm{out}}$ (see Equation (1)). We found an average experimental bulk sensitivity of 4200rad/RIU, which agrees reasonably with the prediction from Equation (1), i.e., $S/I_{\mathrm{out}}=4900\;\mathrm{rad}/\mathrm{RIU}$, for the values given in Table A1. The molecular binding processes that are monitored with photonic biosensors often take place on time scales of seconds or even minutes, so initially a sampling frequency of 50Hz is chosen, with a low pass filter at $B_{d}=25\;\mathrm{Hz}$. The noise was computed as the standard deviation of the extracted phase $\hat{\varphi }$, i.e., $\sigma /I_{\mathrm{out}}$, when only purified water was running over the sensor and was found to be 45mrad (dominated by mechanical noise as described below). Note that the electrical and optical noise in the system do not depend on the specific refractive index change. Under these conditions we obtained an initial LOD of $3\times 10^{-5}\;\mathrm{RIU}$, as shown in Figure 1a.

## 3. Enhancement Strategies

### 3.1. Sampling Frequency and Mechanical Noise

**Figure 4:**
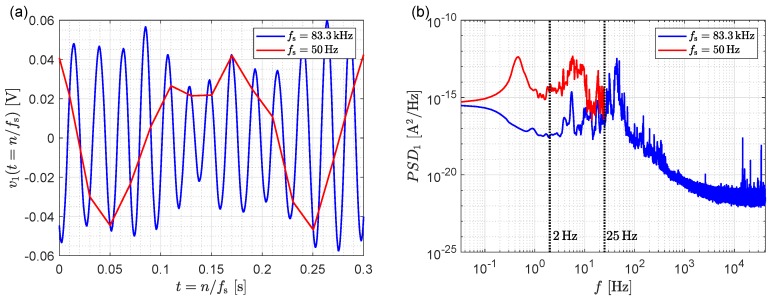
(**a**) Signal of one of the photodiodes at the sensor output in the presence of mechanical vibrations in the setup for a sampling frequency of (a) 50Hz and (b) 83kHz. Only the fast sampling rate reveals the harmonic nature of the oscillations. (**b**) Power spectral density of the photodiode signal. The higher sampling frequency removes the spectral aliasing.

While a comparatively low sampling frequency of 50Hz is reasonable in terms of the time constants involved in molecular binding processes, in terms of noise analysis much higher sampling frequencies are required. This is illustrated in Figure 4a, which shows the noise at one of the electrical outputs of the sensing system in Figure 2 when no sensing is taking place, sampled at 50Hz and at the maximum rate of 83kHz of our DAQ system; the DC component of the signal has been removed for clarity. In the setup a fiber array is used to couple light to/from the chip, therefore mechanical vibrations create micro-misalignments, resulting in a modulation of the power coupled into and out of the chip. While the strength of the modulation is strongly dependent on the specific alignment between fiber array and the chip, its natural frequency is determined by the mechanical construction of the setup. The modulation becomes apparent if a sufficiently fast sampling rate is chosen, revealing, in our case, a mechanical oscillation mode with a natural frequency of ∼50Hz (see blue line in Figure 4a). The mechanical origin of these oscillations was confirmed by placing accelerometers on the fiber array holder and mechanically exciting the alignment stages with a small impact hammer, revealing a vibrational mode around ∼50Hz.

The unilateralized power spectral densities of the noise signal are shown in Figure 4b, referred to the amplifier input, i.e., divided by $G^{2}$. Note that the higher sampling rate yields a reduction in the power spectral density, as spectral aliasing is now avoided. Indeed the noise power $\sigma^{2}$ in the 25Hz bandwidth, computed as the integral of the power spectral densities in Figure 4b, is reduced by a factor ∼188. Assuming a similar reduction is achieved in all three photodiodes, Equation (7) predicts a LOD enhancement of sqrt $\sqrt{188}\approx 13.7$. Sensing experiments carried out with the 83kHz sampling frequency reveal a reasonable match with an enhancement factor ∼8, to a LOD of $3.8\times 10^{-6}\;\mathrm{RIU}$ (see Figure 1a—step I). Furthermore, reducing the filter bandwidth to a more aggressive 2Hz, reduces the noise power by an additional factor 52, which according to Equation (7) should improve the LOD by a factor $\sqrt{52}\approx 7.2$. A measured LOD enhancement of ∼2.5 to $1.5\times 10^{-6}\;\mathrm{RIU}$ has been experimentally determined as illustrated in Figure 1a—step II. The discrepancy between the predicted and experimentally observed LOD enhancements is attributed to the different mechanical noise characteristics of each of the three outputs.

Finally we observe in Figure 4b that the power spectral density drops significantly for frequencies beyond 100Hz, suggesting that mechanical noise is the dominant noise source at low frequencies. For further optimization it is thus critical to dampen these oscillations, which can be achieved, for instance, by improving the mechanical isolation of the setup, or by gluing the input/output fibers to the integrated chip.

**Figure 5:**
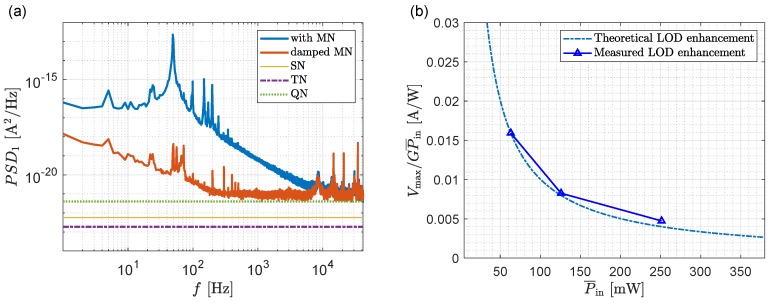
(**a**) Shows the theoretical shot (fine orange), electrical (purple dashed), quantization (green dashed) current power spectral densities, and the measured power spectral densities with mechanical noise and with dampened mechanical noise. (**b**) In the presence of dominant quantization noise the LOD is expected to follow the ratio $V_{\max}/\left(G{\bar{P}}_{\mathrm{in}}\right)$, which is in good agreement with the experimental results.

A separate set of measurements was used to assess the noise reduction that can be achieved through mechanical damping. Figure 5a compares the noise power spectral density when the fiber array is positioned above the chip and when it is brought into direct contact with the chip. A significant reduction, of approximately two orders of magnitude (a factor 100) is achieved. This should provide a further LOD enhancement of $\sqrt{100}=10$ according to Equation (11). Indeed, sensing experiments reveal a LOD enhancement from $1.5\times 10^{-6}$ to $3.5\times 10^{-7}\;\mathrm{RIU}$ (a factor 4.3) as shown in Figure 1a.

### 3.2. Quantization, Shot and Thermal Noise

With the initially dominant mechanical noise dampened, we focus on electrical noise sources, i.e., shot, thermal and quantization noise. Representative values for the relevant setup parameters are $P_{\mathrm{out}}=160\;\mu W$, R=1A/W, $B_{w}=450\;\mathrm{kHz}$, $f_{s}=83\;\mathrm{kHz}$, $\mathrm{NEP}=1.25\;\mathrm{pW}/\sqrt{\mathrm{Hz}}$, $V_{\max}=5\;V$, γ=0.7 and G=31kV/A. Comparison of the noise floor in Figure 5a with the theoretical power spectral densities given by Equations (2)–(4) reveals that the noise level is dominated by quantization noise, with smaller contributions from shot and thermal noise. Taking into account Equations (8)–(10) the basic strategy is to use a high input power to minimize the impact of thermal and shot noise while aiming to use the full range of the DAQ to mitigate quantization noise. We use the following procedure to achieve this:

1. Choose a large value of the DAQ voltage range ($V_{\max}$).
2. Increase the input power ($P_{\mathrm{in}}$) as much as possible without saturating the DAQ, possibly with the help of an external fiber amplifier (EDFA). This helps reduce the impact of shot, thermal and quantization noise.
3. If the DAQ is not close to saturation, increase the gain of the amplifier (*G*). Depending on the specific amplifier, this may reduce the amplifier NEP.
4. If the DAQ is not close to saturation, decrease the DAQ voltage range to minimize quantization noise.

In a situation where the quantization noise is dominant, the above procedure should enhance the LOD according to the change in the ratio $V_{\max}/\left(G{\bar{P}}_{\mathrm{in}}\right)$ (see Equation (10)). As illustrated in Figure 5b, there is indeed a good agreement between this ratio and the experimental LOD improvement that is obtained by increasing the input power. This electrical optimization step improves the LOD from $3.5\times 10^{-7}$ to $7.4\times 10^{-8}\;\mathrm{RIU}$, as shown in Figure 1a.

### 3.3. Residual Baseband Noise

In Figure 5a we observe that the noise power floor at frequencies in the kHz range is still almost three orders of magnitude, i.e., a factor 1000, lower than at baseband, which may be either due to residual mechanical noise or flicker noise in the electronics [40]. While modulating the input signal to kHz frequencies will not reduce any residual mechanical noise (because it is itself a modulation of the signal), it would eliminate flicker noise. However, modulating the signal comes at the cost of lost power: even a perfect amplitude modulation $P_{\mathrm{in}}\left(0.5+0.5\cos\left(\omega_{\mathrm{mod}}t\right)\right)$ only transfers 1/8 of the power to the modulation frequency $\omega_{\mathrm{mod}}$. According to Equation (7), the expected improvement in LOD is therefore reduced to a factor $∼\sqrt{1000/8^{2}}=4$. We used a variable optical attenuator to modulate the input signal to 800Hz, and repeated the LOD experiments, performing a software demodulation of the signal (this could alternatively be done with a lock-in amplifier). We observed an improvement in the LOD from $7.4\times 10^{-8}$ to $3.6\times 10^{-8}\;\mathrm{RIU}$, i.e., a factor ∼2 improvement, indicating that some residual mechanical noise is still present. Indeed, a simple sinusoidal model for the mechanical oscillations [41] shows that for our experimental conditions movements of the order of only ∼150nm limit the LOD to the observed values.

Finally, in this situation an additional improvement in LOD of a factor ∼2.6 can be observed by increasing the integration time of the digital filter from 0.5s to 5s, specifically from $3.6\times 10^{-8}$ to $1.4\times 10^{-8}\;\mathrm{RIU}$. A comparison of our results with a selection of state-of-the-art sensors is shown in Table 2, revealing that with effective noise treatment even waveguides with a comparatively poor sensitivity like ours ($S_{\mathrm{wg}}∼0.2\;\mathrm{RIU}/\mathrm{RIU}$) can yield excellent LOD values.

We note that if the mechanical oscillations were completely dampened (e.g., by integrating the photodiodes into the chip), the theoretical analysis presented in [8] predicts that an LOD below $10^{-9}\;\mathrm{RIU}$ should be attainable for our experimental conditions, when quantization noise is considered as described in Appendix A.1. Even better LODs are possible by further increasing the input power, using lower noise electronics or employing a specialized DAQ with more than 16 quantization bits, opening exciting prospects towards ultra-low detection limits.

## 4. Conclusions

Different types of read-out noises can have a significant impact on the limit of detection of photonic biosensors. We have shown that by identifying mechanical and electrical noise sources, and systematic fine-tuning of readily adjustable system parameters, such as sampling rate, DAQ range, input power, and amplifier settings, as well as adequate averaging, LOD enhancements of several orders of magnitude can be achieved. Indeed, using an interferometric sensing chip, with comparatively low sensitivity waveguides, our optimization strategy yields a limit of detection of ∼$10^{-8}\;\mathrm{RIU}$. We are confident that our approach can be readily extended to other biosensing architectures, paving the way for integrated sensors with ultra-low limits of detection.

## Figures and Tables

**Table 1:** Refractive index details of the different injected NaCl solutions at a wavelength of 1.55μm.

Solution	n[RIU]	Δn[RIU]
Purified Water (Milli-Q)	1.3162	-
NaCl 0.5M (3% Mass Perc.)	1.3211	$4.9\times 10^{-3}$
NaCl 1.0M (6% Mass Perc.)	1.3262	$1.0\times 10^{-2}$
NaCl 1.5M (9% Mass Perc.)	1.3313	$1.5\times 10^{-2}$
NaCl 2.0M (12% Mass Perc.)	1.3466	$2.0\times 10^{-2}$

**Table 2:** Comparison of bulk LOD values achieved with recent resonant and interferometric photonic sensors.

Type	Reference	Year	LOD[RIU]
Ring Resonator	[24]	2011	$1.6\times 10^{-5}$
Ring Resonator	[16]	2016	$2\times 10^{-6}$
Ring Resonator	[17]	2016	$3.9\times 10^{-4}$
Ring Resonator	[14]	2017	$3.7\times 10^{-4}$
Ring Resonator	[22]	2017	$2.4\times 10^{-6}$
Ring Resonator	[23]	2017	$8.5\times 10^{-7}$
MZI	[32]	1998	$5\times 10^{-6}$
MZI	[26]	2012	$1.9\times 10^{-7}$
MZI	[11]	2013	$5.4\times 10^{-6}$
MZI	[18]	2016	$5\times 10^{-7}$
MZI	[21]	2017	$8.8\times 10^{-7}$
MZI	[22]	2017	$2.7\times 10^{-8}$
MZI	[4]	2018	$3\times 10^{-6}$
MZI	[27]	2018	$4.7\times 10^{-7}$
MZI	[31]	2019	$3.7\times 10^{-7}$
MZI	This work	2019	$1.4\times 10^{-8}$

## Supplementary Materials

The following is available online at https://www.mdpi.com/1424-8220/19/17/3671/s1: experimental data (Figure 1, Figure 4, Figure 5a) is available as *.mat or *.csv file and is accessible with the Matlab software [42] via the provided Matlab scripts (*.m).

## Appendix A

### Appendix A.1. Quantization Noise

Quantization noise results from the digitalization process of the data acquisition board (DAQ) [35]. The continues signal v(t) is sampled at $t_{n}=n/f_{s}$, and its value is quantized resulting in the digital signal v[n]. The quantization step depends on the number of bits, *M*, of the DAQ, and the range of input voltages $\left[-V_{\max},+V_{\max}\right]$:

(A1) $$\Delta v_{\mathrm{qs}}=\frac{2V_{\max}}{2^{M}}.$$

The noise added at the input of the TIA can be formulated as

(A2) $$\sigma_{\mathrm{QN}}^{2}=\frac{\gamma^{2}}{G^{2}}\Delta v_{\mathrm{qs}}^{2}\frac{2}{f_{s}}B_{d}={\mathrm{PSD}}_{\mathrm{QN}}B_{d},$$

where γ is the unitless quality factor of the DAQ, the factor $1/G^{2}$ results from the transfer of the noise from the output of the DAQ to the input of the TIA, while the factor $2/f_{s}$ stretches the noise over the Nyquist-spectrum.

### Appendix A.2. Equipment Parameters

The configuration parameters of the equipment used in the setup in Figure 2 are given in the table below.

**Table A1:** Exemplary equipment and parameters for the setup in Figure 2.

Parameter	Value	Unit
*Laser - WSL-100 (Santec)*		
$P_{0}$	[7…15.5]	dBm
$\lambda_{0}$	1.55	μm
*EDFA - EAD-500C (IPG Photonics)*		
$P_{\mathrm{output}}$	[16.5…27]	dBm
*Photo Detection - PDA10CS-EC (Thorlabs)*		
*R*	1.05	A/W
*G*	[0.99,3.12,9.94,31.33,95.86]	kV/A
NEP(G)	[60,10,3,1.25,1.4]	$\mathrm{pW}/\sqrt{\mathrm{Hz}}$
$B_{w}$	450	kHz
*DAQ - NI USB-6210 (National Instruments)*		
$f_{s,\max}$	250	kHz
γ	0.7	–
*M*	16	–
$V_{\max}$	[0.2,1.0,5.0,10.0]	V
$f_{s}$	$f_{s}\leq f_{s,\max}/3$	kHz
*General*		
${\bar{\eta }}^{2}$	≈1/100	–
$L_{s}$	6	mm
$S_{\mathrm{wg}}$	0.2 (simulated [38])	RIU/RIU
